# Genome-Wide Analysis of Gene Expression Provides New Insights into Waterlogging Responses in Barley (*Hordeum vulgare* L.)

**DOI:** 10.3390/plants9020240

**Published:** 2020-02-13

**Authors:** Ana Borrego-Benjumea, Adam Carter, James R. Tucker, Zhen Yao, Wayne Xu, Ana Badea

**Affiliations:** 1 Brandon Research and Development Centre, Agriculture and Agri-Food Canada, 2701 Grand Valley Road, Brandon, MB R7A 5Y3, Canada; ana.borrego@canada.ca (A.B.-B.); adamcarterd28@gmail.com (A.C.); james.tucker@canada.ca (J.R.T.); 2 Morden Research and Development Centre, Agriculture and Agri-Food Canada, 101 Route 100, Morden, MB R6M 1Y5, Canada; zhen.yao@canada.ca (Z.Y.); wayne.xu@canada.ca (W.X.)

**Keywords:** barley, waterlogging stress, RNA-Seq, differentially expressed genes, transcription factors

## Abstract

Waterlogging is a major abiotic stress causing oxygen depletion and carbon dioxide accumulation in the rhizosphere. Barley is more susceptible to waterlogging stress than other cereals. To gain a better understanding, the genome-wide gene expression responses in roots of waterlogged barley seedlings of Yerong and Deder2 were analyzed by RNA-Sequencing. A total of 6736, 5482, and 4538 differentially expressed genes (DEGs) were identified in waterlogged roots of Yerong at 72 h and Deder2 at 72 and 120 h, respectively, compared with the non-waterlogged control. Gene Ontology (GO) enrichment analyses showed that the most significant changes in GO terms, resulted from these DEGs observed under waterlogging stress, were related to primary and secondary metabolism, regulation, and oxygen carrier activity. In addition, more than 297 transcription factors, including members of MYB, AP2/EREBP, NAC, WRKY, bHLH, bZIP, and G2-like families, were identified as waterlogging responsive. Tentative important contributors to waterlogging tolerance in Deder2 might be the highest up-regulated DEGs: Trichome birefringence, α/β-Hydrolases, Xylanase inhibitor, MATE efflux, serine carboxypeptidase, and SAUR-like auxin-responsive protein. The study provides insights into the molecular mechanisms underlying the response to waterlogging in barley, which will be of benefit for future studies of molecular responses to waterlogging and will greatly assist barley genetic research and breeding.

## 1. Introduction

Barley (*Hordeum vulgare* L.) is one of the most important crop species in the world, mainly used as feed for livestock and malt. Based on production, it is placed fourth worldwide as well in Canada [1,2]. Canada is the fourth largest barley producer and the second largest malt exporter in the world. On average, each year approximatively $1 billion is directly generated from the export of feed barley and malt [3]. 

Barley production is affected by biotic pathogens such as fungi, viruses, nematodes, and bacteria, as well as abiotic stresses such as temperature (high and low temperature), water (drought and waterlogging), etc. Barley is more susceptible to waterlogging stress than other cereals, causing chlorosis, degradation of RNA and protein, reduction of nitrogen and other nutrients content in shoots, reduction of shoot and root growth, reduction of leaf area and biomass, and grain yield [4,5,6,7]. Waterlogging as an abiotic stress causes significant grain yield losses that vary from 10% to 50%, or even beyond, depending on the sensitivity of the genotype, depth and duration of flooding, plant developmental stage, temperature, and soil type [4,5,8,9,10]. Waterlogging mainly results from prolonged rainfall and poor soil drainage and has increased in frequency and intensity over the past 60 years worldwide [4,11]. In western Canada, excess moisture has been identified as a main problem for the crops grown, including barley. In the recent years the crop claims due to excess moisture significantly increased in this region [12]. 

The physiological effect of waterlogging has been investigated in many crop species. Waterlogging causes excessive moisture in soil, under which the diffusion of gases is reduced, decreasing water and nutrient absorption by roots [13]. Waterlogging occurs with either partial (hypoxia) or complete (anoxia) depletion of oxygen in the soil, increasing crop yield losses [11,13,14]. It is known that plants have developed two main strategies to adapt to waterlogging stress: (1) oxygen deficiency avoidance by morpho-anatomical modifications; and (2) adaptation to oxygen deficiency by metabolic modifications [15]. To cope and survive for a certain period of time under low oxygen concentrations, plants possess various metabolic adaptations. Energy metabolism is the first to be affected by oxygen deficiency, involving a shift from oxidative phosphorylation to anaerobic fermentation by the activation of the glycolytic pathway to increase adenosine triphosphate (ATP) production. Consequently, to provide nicotinamide adenine dinucleotide (NAD^+^) to maintain glycolysis, the ethanol fermentation pathway, via pyruvate decarboxylase (PDC) and alcohol dehydrogenase (ADH), and the lactate fermentation pathway, via lactate dehydrogenase (LDH), are induced [11,16,17]. Carbohydrate metabolism is also affected by root hypoxia, leading to a differential expression of several proteins, such as enzymes related to starch biosynthesis (ADP-Glucose pyrophosphorylase (AGPase)) and sucrose metabolism (sucrose synthase (SUS), sucrose phosphate synthase (SPS), and invertase (INV)) [18,19,20]. 

Waterlogging stress, among other abiotic stresses, produces reactive oxygen species (ROS) and excessive ROS can cause irreversible oxidization of lipids and proteins, leading to membrane injury. In wheat, waterlogging induces ROS synthesis and ethylene production and inhibits root growth and nutrient and water transport [21]. It was shown that in wheat, waterlogging treatments resulted in the accumulation of ROS in the cortical cells, which were the zone for aerenchyma development [22]. The aerenchyma provides a low-resistance pathway for the transport of oxygen from the shoot to the root apex. In barley, like in other waterlogging-susceptible crops, aerenchyma is formed after waterlogging stress. The faster it is formed the higher the chances of survival are [23]. To overcome ROS injury, plants utilize ROS scavengers, such as glutathione S-transferase (GST), peroxidase (POD), and superoxide dismutase (SOD) [24,25]. SOD action results in the formation of H_2_O_2_ and O_2_; the H_2_O_2_ produced is then scavenged by catalase (CAT) and a variety of PODs. CAT dismutates H_2_O_2_ into H_2_O and O_2_, whereas POD decomposes H_2_O_2_ by oxidation of co-substrates, such as phenolic compounds and/or antioxidants [26]. Ascorbate peroxidase (APX) plays a vital role in plant defense against oxidative stress by catalyzing the conversion of H_2_O_2_ to H_2_O. Glutathione reductase (GR) plays a significant role in maintaining the ascorbic acid and glutathione redox state under oxidative stress. GST provide protection against oxidative stress induced by abiotic stresses and oxidants. Functioning as glutathione peroxidase and dehydroascorbate reductase (DHAR), plant GSTs can catalyze the reduction of hydroperoxides to less harmful alcohols and safeguard protein function from oxidative damage and maintain redox homeostasis by regenerating ascorbic acid from DHA [27]. Ethylene has an important role in the response of plants to biotic and abiotic stresses and is a major regulator of several waterlogging-adaptive plant traits, i.e., submergence–adaptive responses, resistance to hypoxia stress via an ethylene-controlled pathway, and formation of aerenchyma and adventitious roots [28]. The ethylene-responsive element binding factor (ERF) proteins are key transcriptional regulators in response to diverse biotic and abiotic stresses in plants.

In recent years, a large number of quantitative trait loci (QTLs) for waterlogging tolerance affecting important traits have been identified in barley, such as root aerenchyma formation [29,30], root membrane potential [31], root porosity [30,32], and ROS formation [33]. Despite the numerous QTL studies conducted to date on waterlogging in barley, the responsible genes underlying the response to waterlogging remain unknown [34]. 

The RNA-Sequencing (RNA-Seq) approach has been successfully used to interpret the responses to waterlogging stress in multiple crops, such as rice [35], maize [36], poplar [37], cotton [38], soybean [39], sesame [40], and cucumber [41]. The common gene expression responses under low oxygen conditions across these crops involved up-regulation of ethylene biosynthesis, carbohydrate and energy metabolism, nitrogen metabolism, as well as glycolysis and fermentation pathways. The down-regulation of genes associated with the synthesis of cell walls, flavonoids, and amino acids has also been observed (reviewed in Najeeb et al. [42]). 

In barley, limited information is available about the transcriptional response to waterlogging stress. Recently, the gene expression of three different genes in barley genotypes with contrasting responses to waterlogging was assessed by qRT-PCR [43]. These three genes, endotransglycosylase, respiratory burst oxidases, and *PDC*, were previously known to be involved in aerenchyma formation and energy metabolism upon waterlogging stress. Significant up-regulation of these genes in the roots under waterlogging stress of the barley genotypes tested was observed, with the tolerant genotypes showing higher expressions than the sensitive genotypes. Furthermore, a proteomic analysis was used to explore the mechanisms involved in the responses of two barley genotypes with contrasting responses to waterlogging stress [44]. It was found that three candidate genes, *PDC*, *ACO*, and *GST*, were up-regulated in the roots of both genotypes in response to the waterlogging stress, but more induced in the tolerant genotype. 

Development of barley cultivars with resistance to abiotic stresses, including waterlogging, is one of the main barley breeders’ goals. Understanding of the genes that respond to waterlogging would help in development of tolerant barley. Although expression profiles in response to waterlogging stress could vary in different tissues (i.e., root, leaf, etc.), in our study we focused on gene expression in response to waterlogging treatment in barley roots, given their importance in response to this stress, by using the RNA-Seq approach. Two barley genotypes were examined to understand the tolerance mechanisms and signaling pathways that are related to waterlogging tolerance. Our results provide insights into the molecular mechanisms underlying the response of barley to waterlogging stress, which will benefit future barley genetic research and breeding.

## 2. Results

### 2.1. Differentially Expressed Genes of Moderately-Tolerant Barley at 72 h of Waterlogging

**Figure 1:**
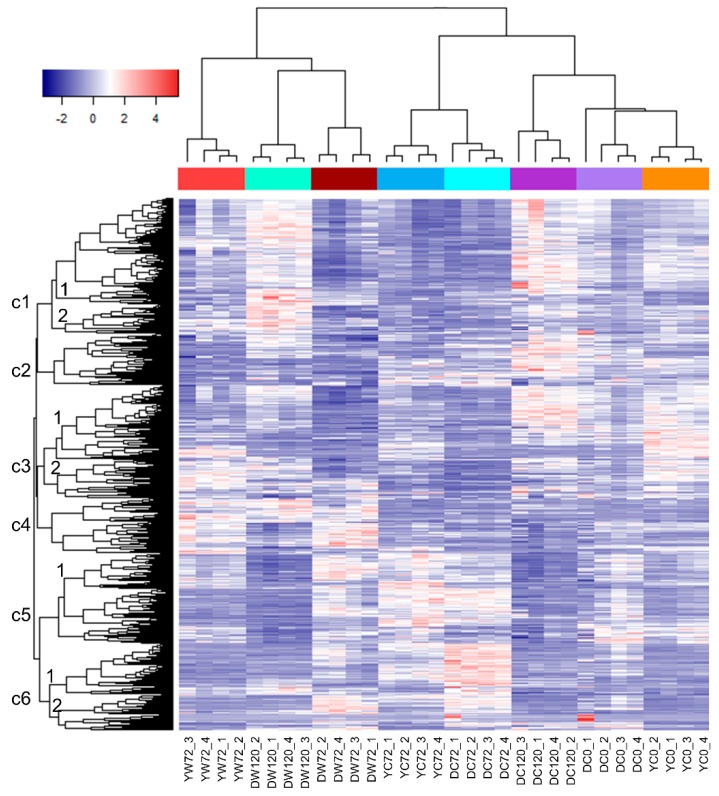
Cluster analysis of the genome-wide gene expression profiles of barley roots under waterlogging and control. Gene expression values (FPKM, fragments per kb transcript length per million reads of library size) were centralized by row using the Heatmap3 program. The genes were clustered by distances. The color scale in the above heatmap shows the expression level; blue indicates low transcript abundance while red indicates high abundance. YC0, Yerong 0 h control; YC72, Yerong 72 h control; YW72, Yerong 72 h of waterlogging; DC0, Deder2 0 h control; DC72, Deder2 72 h control; DC120, Deder2 120 h control; DW72, Deder2 72 h waterlogging; DW120, Deder2 120 h waterlogging.

Six gene expression clusters were observed among eight groups of samples (Figure 1). Yerong at 0 h control had a different overall gene expression profile from Yerong at 72 h control samples. The Cluster 3 genes were more highly expressed in 0 h control than in the 72 h control, but Cluster 5 genes were more highly expressed in Yerong 72 h control than in the 0 h control. After 72 h of waterlogging, a portion of the same Cluster 3 genes remained highly expressed as in the 0 h control, but Cluster 4 genes were more highly expressed in 72 h waterlogging. Interestingly, the highly expressed Cluster 6 at 72 h control was not shown in 72 h waterlogging. This indicates that the 72 h waterlogging lagged most of the gene activities at the original 0 h levels, and inhibited many new activities that should come out at 72 h development.

**Figure 2:**
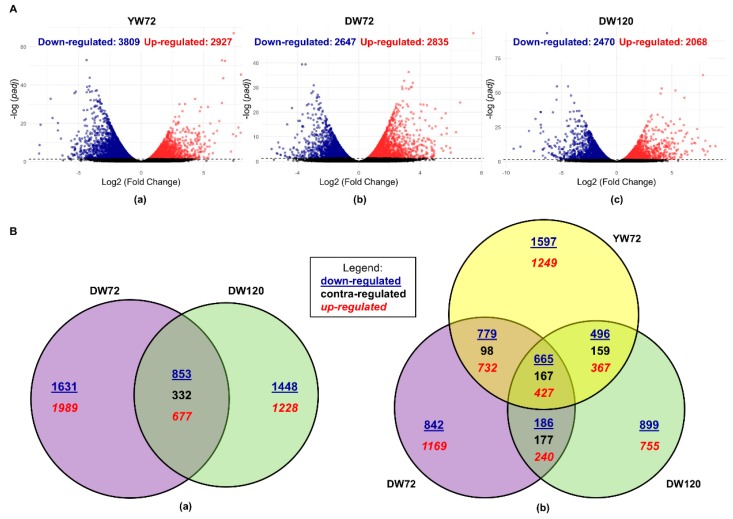
Differentially expressed genes (DEGs) under waterlogging: (**A**) Volcano plot illustrating number of DEGs between flooded and control treatments of Yerong at 72 h (**a**), and Deder2 at 72 h (**b**) and 120 h (**c**). (**B**) Venn diagram illustrating unique and common DEGs in barley roots: (**a**) number of DEGs in Deder2 at 72 and 120 h of waterlogging stress; (**b**) number of unique and shared DEGs between Yerong at 72 h, and Deder2 at 72 and 120 h post waterlogging treatment. YW72, Yerong 72 h of waterlogging; DW72, Deder2 72 h waterlogging; DW120, Deder2 120 h waterlogging.

We next analyzed the 72 h waterlogging and 72 h control data. A total of 6736 differentially expressed genes (DEGs) were identified in this pairwise comparison of Yerong waterlogging vs. Yerong control (3809 down-regulated and 2927 up-regulated) in response to 72 h of waterlogging (Figure 2A(a)). 

### 2.2. Differentially Expressed Genes of Tolerant Barley at 72 and 120 h of Waterlogging

In the control condition without waterlogging stress, Deder2 at 0 h did not show extremely highly expressed clusters, but a highly expressed Cluster 6 at 72 h and highly expressed Clusters 1, 2, and 3 at 120 h (Figure 1). After waterlogging at 72 h, Cluster 4 and sub-Clusters 5-1 and 6-1 were highly expressed, which had a distinct profile from the 72 h control. Interestingly, after 120 h of waterlogging, the same sub-Cluster 1-1 genes were highly expressed, similar to the 120 h control, but sub-Cluster 1-2 genes were highly induced and sub-Cluster 2-1 genes were suppressed. 

The pairwise comparison of Deder2 waterlogging vs. control at the same time-points was performed. In response to 72 h of waterlogging, 5482 DEGs were identified (2647 down-regulated and 2835 up-regulated) (Figure 2A(b)). Furthermore, a total of 4538 DEGs (2470 down-regulated and 2068 up-regulated) were identified at 120 h (Figure 2A(c)). When comparing DEGs at two time-points in Deder2, 1862 DEGs were found to be common between 72 and 120 h of waterlogging (Figure 2B(a)). Additionally, 3620 DEGs (1631 down-regulated and 1989 up-regulated) were only identified at 72 h, and 2676 DEGs (1448 down-regulated and 1228 up-regulated) were unique at 120 h. In this comparison, the time-point 72 h had more DEGs than the time-point 120 h during the waterlogging (Figure 2B(a)). 

The top genes with the highest and lowest log_2_ fold change (logFC) across the two time-points in Deder2 roots were identified (Table S1). The expression of the highest up-regulated DEGs increased with the duration of the waterlogging stress, showing the greatest expression at 120 h: Trichome birefringence-like 19 (8.47 logFC), α/β-Hydrolases superfamily protein (8.23 logFC), Xylanase inhibitor proteins (7.90 logFC), MATE efflux family protein (7.38 logFC), serine carboxypeptidase-like 51 (7.00 logFC), and SAUR-like auxin-responsive protein family (6.83 logFC). Conversely, the most differentially expressed down-regulated genes were Copalyl diphosphate synthase 2 (−7.34 logFC), O-methyltransferase family protein (6.84 logFC), and Dehydrin (−5.68 logFC), whose expression gradually decreased with waterlogging duration.

### 2.3. Differentially Expressed Genes of Moderately-Tolerant and Tolerant Barleys under Waterlogging

Overall, Yerong 0 h control and Deder2 0 h control had a similar expression profile where they were clustered in the same sample sub-cluster (Figure 1). At 72 h without waterlogging, these two lines also had a similar gene expression profiles, although sub-Cluster 6-1 was higher expressed in Deder2 at 72 h control (Figure 1). After waterlogging, Yerong showed commonly induced Cluster 3 and 4 genes at 72 h with Deder2 at 72 and 120 h (Figure 1). Interestingly, Yerong at 72 h had a different Cluster 1 profile than Deder2 at 120 h, which represented a common signature for Deder2 (control and waterlogging at 120 h), and had a different sub-Cluster 6-2 profile than Deder2 at 72 h, which also represented a common signature for Deder2 (control and waterlogging at 72 h).

In total, 2868 DEGs were shared between Yerong and Deder2 at 72 h (Figure 2B(b)). Additionally, 3868 DEGs were uniquely identified in Yerong and 2614 DEGs were uniquely identified in Deder2 (Figure 2B(b)). This illustrates that while Yerong and Deder2 have some shared gene expression responses to waterlogging, there are differences that could represent unique mechanisms of tolerance in each of those genotypes.

### 2.4. Validation of Differentially Expressed Genes 

To validate whether the digital gene expression results were reliable, the DEGs identified in the waterlogged roots were validated through a qRT-PCR assay. The gene *ErTF1* was down-regulated while the *GluD1*, *Hg1*, *WAT1*, and *XEH2* genes were up-regulated in Yerong at 72 h and Deder2 at 72 and 120 h in the waterlogged samples. The expression pattern in the qRT-PCR assay demonstrated that the genes were expressed in a manner consistent with the RNA-Seq results, indicating that the RNA-Seq data were valid (Figure S1). 

### 2.5. Gene Ontology Term Enrichment for All Differentially Expressed Genes at 72 and 120 h of Waterlogging 

The Gene Ontology (GO) term enrichment analyses on all DEGs were performed between the waterlogging and control groups. In genotype Deder2 at 72 h of waterlogging, the main functional groups of DEGs included “metabolic process”, “cellular process”, and “biological regulation” in the “Biological Processes” category; “extracellular region” and “cell part” in the “Cellular Component” category; “catalytic activity”, “transcription regulator activity”, and “binding” in the “Molecular Function” category. At 120 h of waterlogging, the dominant functional groups of the classified genes included “cellular process”, “metabolic process”, “localization”, and “response to stimulus” in the “Biological Processes” category; “membrane” and “extracellular region” in the “Cellular Component” category; and “catalytic activity”, “binding” and “transporter activity” in the “Molecular Function” category.

**Figure 3:**
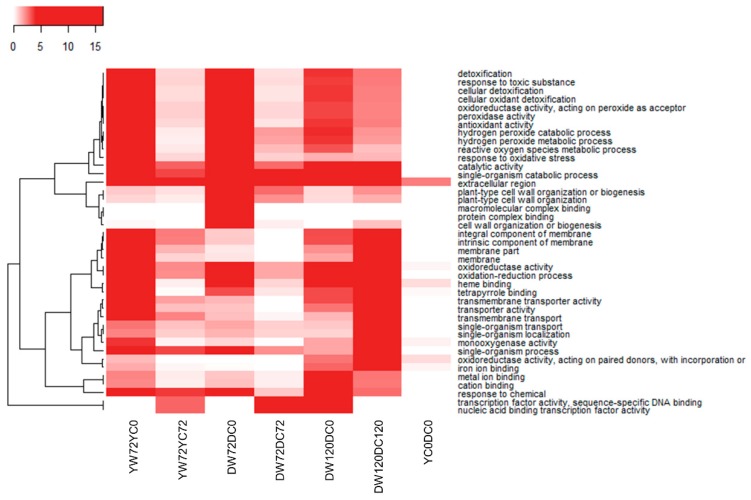
Gene Ontology (GO) enrichment analysis. The GO enrichment analyses were performed on waterlogging group against controls. The adjusted *p*-values of the enrichment significant were transformed by –log10 and clustered by GO terms. A darker color indicates greater significance. YW72YC0, Yerong 72 h waterlogging against 0 h control; YW72YC72, Yerong 72 h waterlogging against 72 h control; DW72DC0, Deder2 72 h waterlogging against 0 h control; DW72DC72, Deder2 72 h waterlogging against 72 h control; DW120DC0, Deder2 120 h waterlogging against 0 h control; DW120DC120, Deder2 120 h waterlogging against 120 h control; YC0DC0, Yerong 0 h control against Deder2 0 h control.

Figure 3 illustrates the most enriched GO terms for multiple comparisons between treatment groups. Some of the top enriched Biological Process GO terms include “hydrogen peroxide metabolic process”, “detoxification”, “oxidation-reduction process”, and “response to oxidative stress” (Figure 3). Other commonly enriched GO terms include “transcription factor activity, sequence specific binding”, “plant-type cell wall organization”, and “extracellular region” (Figure 3). These GO terms represent generally enriched terms over multiple treatment comparisons.

### 2.6. Gene Ontology Term Enrichment of Down and Up-Regulated Genes at 72 and 120 h of Waterlogging

**Figure 4:**
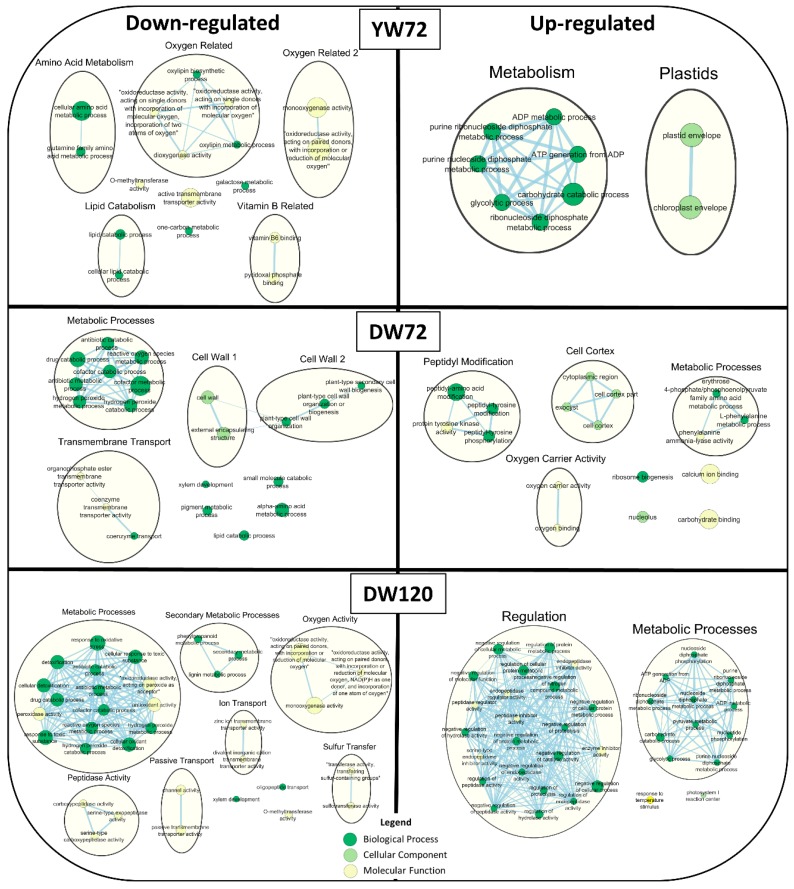
Summary of enriched Gene Ontology (GO) terms of down-regulated (left side) and up-regulated (right side) genes in waterlogged roots of Yerong at 72 h (YW72), as well as Deder2 under waterlogging stress at 72 h (DW72) and 120 h (DW120). Circle size represents number of genes in the GO term, and connections among circles represent overlapping gene sets of each GO term. Larger clusters are generally named based on contained GO terms. GO terms containing more than 500 genes were removed.

To look closer at the enriched GO terms within each genotype and time point, GO term enrichment analysis was performed on down- and up-regulated DEG groups. Many enriched down-regulated GO terms for Yerong at 72 h were identified, including terms related to amino acid metabolism, oxygen related processes, lipid catabolism, vitamin B, and some unique terms including the molecular function GO term “active transmembrane transporter activity” (Figure 4). The enriched up-regulated GO terms included general metabolism functions, such as “ATP generation from ADP” and “glycolytic process”, as well as two plastid-related GO terms “plastid envelope” and “chloroplast envelope” (Figure 4).

Down-regulated GO terms for Deder2 at 72 h included those involved with general metabolic processes, transmembrane transport, the cell wall, and unique GO terms, including “xylem development”, “pigment metabolic processes”, and “lipid catabolic process” (Figure 4). Enriched up-regulated GO terms were varied, and included GO terms related to peptidyl modification, cell cortex, general metabolic processes, oxygen carrier activity, and others such as “nucleolus”, “ribosome biogenesis”, “calcium ion binding”, and “carbohydrate bonding” (Figure 4). 

Enriched down-regulated GO terms in Deder2 at 120 h include metabolic processes like “response to oxidative stress”, “cellular detoxification”, “antioxidant activity”, and “phenylpropanoid metabolic process” (Figure 4). Observed also, were GO terms related to oxygen activity, ion and passive transport, peptidase activity, sulfur transfer, and the ungrouped GO terms of “oligopeptide transport”, “0-methyltransferase activity”, and “xylem development”, which was present at 72 h as well (Figure 4). The enriched up-regulated GO terms encompass many regulatory terms, including “peptidase inhibitor activity” and a class of terms representing negative regulation of a host of processes, including hydrolase and peptidase activity (Figure 4). General metabolic processes similar to those up-regulated in Yerong at 72 h were also observed, including “ATP generation from ADP” (Figure 4). Two ungrouped GO terms, “response to temperature stimulus” and “photosystem I reaction center”, were also identified (Figure 4). 

### 2.7. Expression of Genes Involved in Energy-Consuming Biosynthesis and Metabolism 

Based on the GO enrichment analyses, many DEGs are associated with biosynthesis and metabolism. We found that 21 DEGs (12 down- and 9 up-regulated) in Yerong and 18 DEGs (8 down- and 10 up-regulated) in Deder2 were enriched in the starch and sucrose metabolism pathway (Table S2). Under waterlogging treatment, the expression of the acid β-fructofuranosidase gene, an invertase that hydrolyzes sucrose into glucose and fructose, decreased, while *α-amylase*, *SUS*, *SPS*, phosphofructokinase, and fructose-bisphosphate aldolase gene expression was induced, playing key roles in primary metabolism and plant development (Table S2). Additionally, waterlogging stress increased the abundance of a α-amylase/subtilisin inhibitor transcript by 7.94-logFC in Yerong at 72 h of waterlogging, and 4.10 and 5.42 logFC in Deder2 at 72 and 120 h, respectively.

The glycolysis/fermentation pathway was significantly induced by waterlogging stress. Twenty-eight enriched DEGs (7 down- and 21 up-regulated) in Yerong and 29 enriched DEGs (6 down- and 23 up-regulated) in Deder2 were associated with this pathway. For example, the expression of the glycolysis-related gene NADP-dependent glyceraldehyde-3-phosphate dehydrogenase (*NADP-GADH*) was 2.72 and 3.14-logFC higher in Deder2 at 72 and 120 h of treatment, respectively, while no expression was found in Yerong, and pyruvate kinase (*PK*) was mostly up-regulated in the two genotypes (Table S2). Moreover, alanine aminotransferase (*ALT*) expression, involved in the conversion of pyruvate to *ALT*, was up-regulated by waterlogging in Yerong and Deder2 (Table S2). Consequently, the expression of most of the genes associated with fermentation, *ADH* and *LDH*, were induced in both genotypes, and besides, in Deder2 their expression increased with the duration of the waterlogging stress. However, *PDC*, involved in the first step of ethanol fermentation, was mostly down-regulated (Table S2).

Amino acids also play a role in the waterlogging response in plants. In Yerong at 72 h, the “amino acid metabolism” enriched GO term was down-regulated. For example, the expression of genes related to nitrogen and amino acid metabolism, asparagine synthetase (*AS*), glutamine synthetase (*GS*), glutamate synthase (*GOGAT*), and high affinity nitrate transporters, were down-regulated in both genotypes (Table S2).

### 2.8. Hormone Related Genes and Transcription Factor Genes

Waterlogging influenced stress-induced hormone-related genes that showed contrasting responses to waterlogging. The 1-aminocyclopropane-1-carboxylic acid (*ACC*) synthase and *ACC* oxidase (*ACO*) genes, involved in ethylene synthesis, were up- and down-regulated in barley roots in both Yerong and Deder2. Auxin-induced protein genes in the Yerong and Deder2 genotypes and gibberellin-oxidase genes in Deder2 were mostly down-regulated. In addition, 17 and 21 genes encoding SAUR-like auxin-responsive protein family were identified in Yerong and Deder2 waterlogged roots, respectively, including 11 and 17 up-regulated genes, respectively (Table S3). 

Many genes involved in ROS detoxification were down-regulated in both genotypes. The expression of the ROS scavenger genes *APX* and *SOD* was induced only in Yerong, whereas *GST* and *POD* scavenger genes had a differential expression in both genotypes (Table S3). Accordingly, the GO enriched terms related to ROS production were downregulated in Deder2 at 72 h and 120 h. 

**Figure 5:**
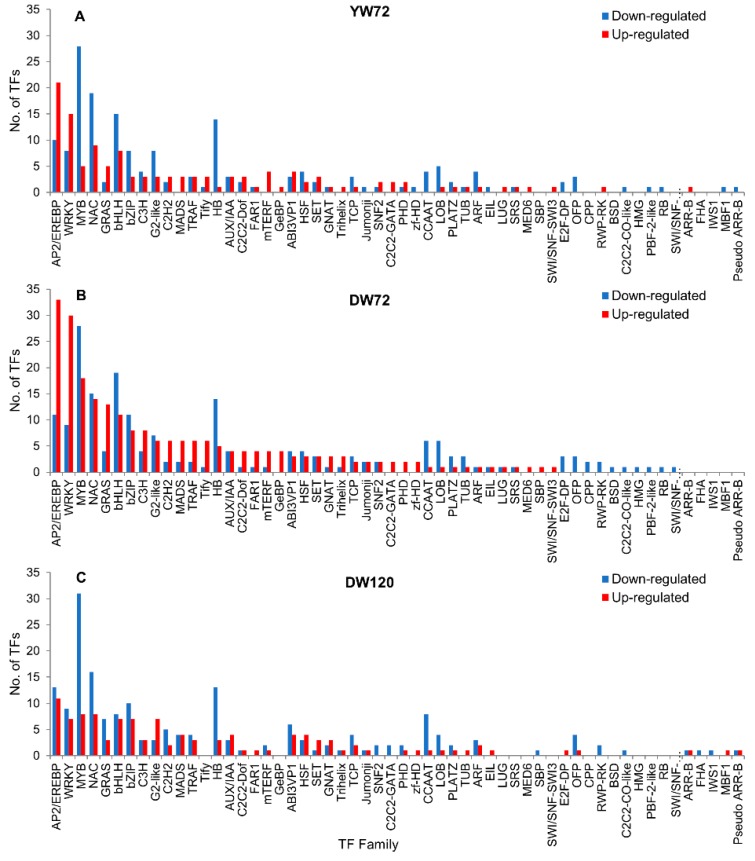
Transcription factors identified in differentially expressed genes in Yerong roots at 72 h (**A**) of waterlogging stress, and Deder2 roots at 72 (**B**) and 120 h (**C**). YW72, Yerong 72 h of waterlogging; DW72, Deder2 72 h waterlogging; DW120, Deder2 120 h waterlogging.

Next, we investigated the TFs associated with waterlogging response. A total of 298 TFs (4.4% of DEGs) were identified in Yerong roots after 72 h under waterlogging stress. A total of 173 TFs were down-regulated, whereas 125 TFs were up-regulated in Yerong after 72 h of waterlogging (Figure 5A). These waterlogging-responsive TFs belonged to 49 families in Yerong at 72 h of waterlogging. Approximately 60% of the TFs in Yerong at 72 h belonged to the families myeloblastosis (MYB) (11% of TFs), APETALA2/Ethylene-responsive Element Binding Proteins (AP2/EREBP) (10%), NAC (9%), WRKY (8%), basic helix–loop–helix (bHLH) (8%), HB (5%), basic leucine zipper (bZIP) (4%), and G2-like (4%). The MYB and EREBP families represented the highest number of significantly expressed TFs at 72 h of waterlogging in Yerong (Figure 5A).

In Deder2 roots after 72 and 120 h under waterlogging stress, respectively, a total of 422 (7.7% of DEGs) and 297 TFs (6.5% of DEGs) were identified. A total of 194 and 185 TFs were down-regulated whereas 228 and 112 TFs were up-regulated at 72 and 120 h of waterlogging, respectively (Figure 5B,C). These waterlogging-responsive TFs belonged to 51 and 44 families in Deder2 at 72 and 120 h, respectively. Approximately 60% of the TFs in Deder2 at 72 and 120 h belonged to the families MYB (11–13% of TFs), AP2/EREBP (8–11%), NAC (7–8%), WRKY (5–9%), bHLH (5–7%), bZIP (4–6%), HB (4–5%), and G2-like (3%). 

The MYB and EREBP families represented the highest number of significantly expressed TFs at 72 h of waterlogging in Yerong and Deder2 roots, while MYB represented the highest number of significantly expressed genes at 120 h of waterlogging in Deder2 roots (Figure 5A–C). Notably, 29 ERFs encoding AP2/EREBP were found to be up-regulated, and the expression of an *ERF-9* gene (HORVU7Hr1G110900) exhibited great up-regulation, presenting increases of 4.91 and 5.84-logFC in Yerong and Deder2, respectively, compared with the control plants (Table S3). 

## 3. Discussion

Though RNA-Seq has been intensively used in studies in crops including barley, we applied for the first time RNA-Seq to investigate the genome-wide gene expression responses of barley to waterlogging abiotic stress. We compared the gene expression in barley roots subjected to 72 h of waterlogging stress in the moderately-tolerant barley genotype Yerong, and 72 and 120 h of waterlogging stress in the tolerant barley genotype Deder2. To the best of our knowledge, this is the first characterization of barley genome-wide gene expression in response to waterlogging stress, and provides new insights into understanding the molecular mechanisms underlying the response to waterlogging in barley.

### 3.1. Global Gene Transcription Changes in Waterlogged Barley Root

A sequencing depth of 45 to 56 million clean reads per library was reached, and approximately 90% of the clean reads were mapped uniquely to the barley genome. This high alignment support that the high confidence genes were considered as being expressed. The gene expression of Yerong at 72 h of waterlogging and Deder2 at 72 and 120 h of waterlogging were compared with those under normal conditions. Our data disclosed significant changes in the gene expression in the roots of the barley seedlings caused by waterlogging. A higher number of DEGs (6736) were identified in Yerong compared to Deder2 under waterlogging conditions, and also a higher number of DEGs were identified at the shorter duration (5482), 72 h, of waterlogging treatment than the longer (4538), 120 h, in Deder2. We found that while Yerong and Deder2 have some shared gene expression responses to waterlogging, there are differences that could represent unique mechanisms of tolerance in each of those genotypes. Many of the genes have shown decreased expression of synthesis pathways, cell wall and secondary metabolism-associated genes, and increased expression of glycolysis, fermentation, and some catabolism pathways, as also shown previously in cotton [38], maize [45], and rice [46]. Overall these results suggest that waterlogging promoted the catabolism of carbohydrates and most proteins induced during waterlogging conditions were enzymes involved in the establishment of the fermentative pathway, indicating that the metabolic switch from aerobic respiration to anaerobic fermentation was a clear response to the stress. 

### 3.2. Waterlogging Stress Up-Regulated Genes Related to Response to Abiotic Stresses

Cell walls are known to be important for protecting cells against pathogens or other environmental factors. The highest up-regulated DEG in Deder2 roots was Trichome birefringence-like 19 gene (HORVU4Hr1G090420), whose underlying function is the ubiquitous modification of cell wall polymers by acetylation and is known to play a structural role in plant growth and microorganism and environmental stresses defenses [47]. This gene encodes the PMR5N domain-containing protein that was reported to play important roles in regulation of transpiration and stress resistance to cold and salt [48]. Another highly up-regulated gene is the α/β-Hydrolases superfamily protein, which is known to be induced by salinity and flooding stress. The α/β-hydrolase gene IbMas was previously found to enhance salt tolerance of the transgenic sweet potato plants by regulating osmotic balance, protecting membrane integrity and photosynthesis, and increasing ROS scavenging capacity [49]. In soybean roots, the α/β-hydrolase superfamily proteins Glyma06g44990 and Glyma12g12800 were increased in response to flooding stress, after a 2-day flooding treatment [50]. The Xylanase inhibitor protein gene encodes the Glyco_hydro_18 domain-containing protein that is involved in osmotic and low temperature stress responses in plants, helping them to survive in stressful environments [51]. The highly up-regulated Serine carboxypeptidase-like protein gene *OsBISCPL1* has a similar domain in rice and was reported to be involved in regulation of defense responses against pathogen infection and oxidative stress [52]. The overexpressed MATE efflux family protein gene is a membrane transporter mediating root signaling and adaptive responses to oxygen deprivation and soil flooding. MATE efflux family protein plays a key role in protecting plants against drought stress [53]. In rice roots, the gene *Os10g034510* from the MATE efflux family protein domain was strongly up-regulated under cadmium (Cd) stress, suggesting the role of this family protein in Cd detoxification via export of Cd from the cytoplasm. The Cd causes oxidative stress to cells, which is also true for other abiotic stresses such as drought, salt, cold, and ABA. Therefore, some similarities in gene regulation may exist among these stress responses [54]. The highly up-regulated gene SAUR-like auxin-responsive protein has been previously reported to play key roles in integrating hormonal and environmental signals into distinct growth and developmental responses [55]. In wheat, SAURs was down-regulated under salt stress and, in *Arabidopsis*, the overexpression of SAURs increased tolerance to drought and salt stress. It is also involved in plant growth and development, up-regulating stress-responsive genes, and inhibiting H_2_O_2_ accumulation and chlorophyll decrease under abiotic stress [56]. The highly down-regulated genes, such as Copalyl diphosphate synthase 2, O-methyltransferase family protein, and Dehydrin, are involved in secondary metabolism. The Copalyl diphosphate synthase 2 gene responds to arsenic detoxification in rice [57]; the O-methyltransferase family protein gene is involved in the methylation of the oxygen atom of a variety of secondary metabolites, including phenylpropanoids, flavonoids, and alkaloids, playing a key role in lignin biosynthesis, stress tolerance, and disease resistance in plants [58]; and the Dehydrin gene has been reported to be involved in the response to cold and drought stress in wheat [59].

### 3.3. Effects on ROS Production

ROS-scavenging enzymes and antioxidants are induced to protect the plants against ROS, which is accumulated under waterlogging conditions creating oxidative stress [60]. They play a critical role, in the plant cells, in the survival under waterlogging of many plants, such as winter wheat [61], maize [45], barley [33], and cucumber [62]. The top 11 Biological Process GO terms for all DEGs were related to ROS catabolic processes, including hydrogen peroxide metabolic process, detoxification, oxidation-reduction process, response to oxidative stress, etc. Our results showed that *SOD* gene expression was increased with waterlogging only in the moderately-tolerant cultivar, Yerong (1.19 logFC), but as expected not in the tolerant Deder2. Zhang et al. [63] reported that SOD enzymatic activity was increased in barley leaves with waterlogging treatment and the barley-sensitive cultivar had higher activity than the tolerant one during the experimental duration. Accordingly, Hwang et al. [64] found that waterlogging led to a higher increase in SOD activity for a waterlogging-sensitive cultivar of sweet potato than for the tolerant one. Therefore, it may be assumed that an increase in SOD activity under waterlogging stress could be indicative of an increased production of ROS. A negative correlation between SOD activity and waterlogging tolerance was reported by Zhang et al. [65]. They suggested that elevated SOD levels might be used as stress markers but not as traits conferring waterlogging tolerance in barley. Zhang et al. [63] found that POD and CAT activities increased in barley leaves at early stage of waterlogging treatment, showing a substantial increase with the progress of waterlogging exposure, in the tolerant cultivar, while decreased in the sensitive one, and GR activity increased in both tolerant and sensitive cultivars. Our results showed that *CAT* expression increased over the waterlogging duration from −0.61 logFC at 72 h to 0.83 logFC at 120 h of waterlogging in Deder2. One *GR* gene was up-regulated in Yerong and two *GR* genes in Deder2 at 120 h, and *APX* was induced in Yerong. Although three *POD* genes were up-regulated, most of them were down-regulated. Yordanova et al. [66] reported that soil flooding affected differently the activity of ROS enzymes in barley leaves. Thus, the 72 to 120 h of soil flooding decreased the activity of SOD, while POD, CAT, and APX activity significantly increased over the flooding time, and the GR activity was insignificantly influenced over the course of treatment. Zhang et al. [65] reported that, in barley leaves, the SOD and CAT activity of waterlogging-tolerant genotypes decreased after seven days of waterlogging treatment, and that there was no effect on APX activity of those genotypes while increased POD activity was found at seven days only in one of the waterlogging-tolerant genotypes tested, which started to decrease afterwards. Lee et al. [67] showed down-regulation of the *CAT* gene and upregulation of *POD*, *SOD*, and *GST* in leaves of rape seedlings under waterlogging stress while Qi et al. [68] proposed that the *POD* gene was upregulated, whereas *SOD*, *CAT*, and *GST* were downregulated under stress. All of these suggest that there might not be a clear, general correlation between waterlogging stress tolerance and the activity of major enzymatic antioxidants, but a more genotype-dependent one. When performing GO enrichment analysis on up and down-regulated genes, ROS related GO terms were down-regulated in Deder2 at 72 and 120 h. For example, the discrepancy between our results and those reported by Gill et al. [33] that screened the barley roots for ROS production under hypoxia stress could be due to the treatment applied (hypoxia solution—0.2% agar vs. waterlogging) but most likely is due to the timing of the measurements, 48 h vs. 72 and 120 h. Previously, Zhu et al. [69] has shown that the activities of SOD, CAT, and POD in grapevine leaves under waterlogging stress increased substantially compared to the control but began declining after 24 h (CAT) and 96 h (SOD and POD). A similar trend was observed in Deder2 for those three *POD* up-regulated genes identified in our study where they were more expressed at 72 h (2.56, 2.00, and 2.21 logFC) compared to 120 h (2.15, 1.48, and 1.47 logFC). We found that ten *GST*-encoding genes were significantly up-regulated by waterlogging stress in Yerong and Deder2, although they were more induced in Deder2, in which most of them increased with waterlogging duration. This result suggested that while many genes involved in ROS were not up-regulated in Yerong and Deder2 at the time points monitored, ROS-scavenging via *GST* could be an important mechanism in their overall resistance to waterlogging stress. A recent study revealed that the expression levels of *GST* were increased in all barley genotypes under waterlogging stress, but greater changes were observed in Yerong, among other tolerant genotypes, suggesting that more efficient ROS detoxification occurs in tolerant genotypes under waterlogging stress [44]. 

### 3.4. Effects on Energy-Consuming Biosynthetic Processes

Upon exposure to waterlogging stress, plants could exhibit a large number of responses at the molecular, cellular, and whole-plant levels, and the metabolic adjustment. Phenylpropanoid biosynthesis has been demonstrated to contribute to various aspects of plant biotic and abiotic responses and synthesis of phenylpropanoid-based polymers, such as lignin and flavonoids [42]. In our study, a number of other biological processes in barley roots were affected by waterlogging. These included regulation of molecular function, cellular metabolic and cellular-wall organization or biogenesis, localization, metabolic process, and response to stimulus. Moreover, several molecular functions were also affected by waterlogging stress, such as antioxidant, binding, catalytic, transporter and transcription regulator activities, as well as the molecular function regulator. Regarding cellular components, the extracellular region, cell part, and membrane were affected by waterlogging stress. Plants can activate additional responses to low oxygen conditions, including the down-regulation of energy consuming processes. Most of the genes involved in the biosynthesis of flavonoids, phenylpropanoids, diterpenoid, isoquinoline alkaloid and cutin, suberine, and wax were down-regulated in the roots of waterlogged barley.

### 3.5. Effects on Carbon Metabolism

Oxygen deficiency in plants due to excess moisture conditions causes energy deprivation, affecting the survival of plants after waterlogging. This is due to oxidative phosphorylation being switched to anaerobic fermentation to maintain ATP production [11]. Our data showed that many genes with potential roles in carbohydrate and energy metabolism were up-regulated. Enzymes such as ADH, PDC, and SUS are all critical for the breakdown of sucrose in glycolysis and subsequent fermentation [70]. The hydrolysis of sucrose into two hexose phosphates can be performed by either of two enzymes, INV or SUS. Upon oxygen deprivation, multiple species increase levels of SUS and repress the activity of INV. INV requires two equivalents of ATP to phosphorylate the two hexoses produced from sucrose [71]. Alternatively, the SUS pathway catalyzes the reversible conversion of sucrose and UDP to UDP-glucose and fructose, yielding UTP, a high-energy molecule that can substitute for ATP, plays a crucial role in providing an adequate sugar supply during anoxic stress [11,72]. In our study, several *INV* genes (a.k.a. acid β-fructofuranosidase) were down-regulated in response to waterlogging stress, whereas the *SUS*-encoding genes were induced in both Yerong and Deder2 and in both time-points for Deder2. These results are in agreement with previous studies in which it is indicated that under hypoxia conditions, the sucrose degradation shifts from INV to SUS. For example, in rice INV activity was depressed and that of SUS was enhanced, with SUS being the enzyme mainly responsible for sucrose breakdown under anoxia [73]. In soybean roots, *INV* genes were down-regulated and *SUS* genes up-regulated in response to hypoxia conditions in both tolerant and sensitive cultivars, in all stress durations [74]. In *Arabidopsis*, similarly to our study, it has been shown that oxygen deprivation increases total SUS activity [75] and also the ethanolic fermentation genes *ADH* [76], indicating that these genes are essential for tolerance to low oxygen. In addition, other genes involved in sucrose and starch metabolism and glycolysis were up-regulated in Yerong and Deder2 roots, such as *α-amylase*, *SPS*, phosphofructokinase and fructose-bisphosphate aldolase, *NADP-GADH*, and *PK*. These results were in agreement with recent studies in tef, a cereal grass [77], and maize [45].

Three main fermentation pathways are active in plants during flooding: ethanol, lactic acid, and a plant-specific pathway, which produces ALT from glutamate and pyruvate, involving ALT [19]. In this study, ALT was induced by waterlogging in Yerong and Deder2 roots. Ethanolic fermentation is the most important fermentative pathway in plants, where there is a carboxylation of pyruvate to acetaldehyde by PDC, and then reduced to ethanol by ADH. Arora et al. 2017 reported that in the maize waterlogging-tolerant genotype tested, the *ADH* gene was differentially expressed at a highest level of 7.6-fold in waterlogging conditions followed by *PDC*. More *PDC* was induced in tolerant barley genotypes vs. sensitive genotypes under waterlogging stress [44]. However, in our study, *PDC* was differentially expressed, not showing a strong up-regulation under waterlogging conditions. This might be due to the much shorter duration of waterlogging treatment in our study (72 and 120 h vs. 3 weeks). Nevertheless, ADH, which is an important anaerobic polypeptide, was also mostly up-regulated in roots of Yerong at 72 h and Deder2 during the 72–120 h period. The lactic acid fermentation pathway involves pyruvate catabolism to lactate by LDH. In our study, *LDH* expression was up-regulated in both genotypes, and for Deder2 also at both time-points. GO enrichment analysis also showed genes involved in glycolysis were up-regulated in Yerong at 72 h and Deder2 at 120 h. These results regarding the carbohydrate metabolism are in agreement with other studies where anaerobic respiration was promoted by waterlogging, as observed by the up-regulation of DEGs encoding enzymes in glycolysis and fermentation, considered key factors in the response to waterlogging in maize [45], soybean [39], and cotton [78]. Interestingly, in our study the ATP synthase expression was down-regulated in Yerong, but there was no effect on the expression in Deder2. In a previous proteomic study in barley, ATP synthase was also down-regulated in Yerong, as in the other barley cultivars, although greater changes were observed in the sensitive genotypes. This indicates that the glycolysis and fermentation pathway was activated to maintain ATP production under stress conditions. As a result, the demand for carbohydrates increased and carbon metabolism significantly increased in waterlogged barley roots. 

### 3.6. Effects on Nitrogen and Amino Acid Metabolism

Nitrogen and amino acids metabolism are deeply affected by oxygen limitation and energy shortage and this was also the case in our study that investigated the changes in the roots of the barley genotypes Yerong and Deder2 in response to the 72 and 120 h waterlogging treatment. Earlier, Ren et al. [79] reported that, in maize leaves, GS and GOGAT activities declined significantly after waterlogging, leading to a significant drop in the activities of N metabolism enzymes and sugar metabolism enzymes, affecting the synthesis and transformation of amino acids. They also reported a down-regulation in *GS* and *GOGAT* genes in barley roots. Moreover, Limami et al. [80] suggested that the major reconfiguration of amino acid metabolism under hypoxia consisted of a concerted modulation of nitrogen flux through the pathways of both alanine and glutamate synthesis in *Medicago truncatula*. The ATP-consuming enzymes GS and AS were significantly inhibited, probably as part of a cellular strategy to mitigate the damaging effect of the energy crisis. Kreuzwieser et al. [37] proposed that hypoxia inhibits the TCA cycle and activates the glycolysis and fermentation pathways, resulting in the accumulation of amino acids closely derived from glycolysis intermediates. In cotton leaves, a reduction in the expression of nitrogen metabolism-related genes was also reported as a response to waterlogging [78]. Similarly, in our study, most of the accumulating amino acids were closely derived from pyruvate (e.g., ALT), while the decreasing amino acids (e.g., GS, AS) were mostly derived from TCA cycle intermediates. 

### 3.7. Transcription Factors Responses to Waterlogging

Transcription factors have been suggested to play important roles in abiotic stress-induced gene regulation network. Currently, the known transcription factors that respond to waterlogging stress include the bZIP, NAC, WRKY, MYB, ERF, and bHLH families. Within the barley genome, we identified more than 297 TFs as waterlogging responsive, and a high number of TFs in both Yerong and Deder2, and in both time-points for Deder2, that were up-regulated by waterlogging stress. The majority of the TF genes belonged to the MYB, AP2/EREBP, NAC, WRKY, bHLH, bZIP, HB, and G2-like families. Among these families, AP2/EREBP and MYB represented the highest number of significantly expressed TFs under waterlogging stress, consistently with various studies that have shown that these TF families are involved in abiotic stress and they positively improve plant tolerance [81,82]. AP2/EREBP has important regulatory functions in environmental stress tolerance. The overexpression of the OsEREBP1 gene confers drought-stress tolerance in transgenic rice [83]. In our study, the *ERF-9* (HORVU7Hr1G110900) gene up-regulated in the roots of Yerong and Deder2 (4.91 and 5.84 logFC, respectively) contains the AP2 superfamily involved in stress tolerance. In rice, the gene SUB1B (LOC_Os09g11480) with a similar domain is one of the three ERF factors within the major QTL Submergence tolerance 1 (*SUB1*) [84]. The *SUB1B* gene function is unknown, but it is a member of the rice VII ERF family, which includes several proteins involved in tolerance to hypoxia or avoidance of hypoxia [11]. In rice roots, the expression of TFs genes containing WRKY, AP2 (LOC_Os09g11480), and MYB domains was greatly enhanced during radial oxygen-loss barrier formation, and it was suggested that these TFs are involved in regulating suberin biosynthesis in the outer part of the roots during radial oxygen-loss barrier formation [85]. Short term waterlogging in two maize lines revealed the up-regulation of *Zm-microRNA172*, which targets the repression of the AP2/ERF TFs ZM5G862109 and ZM2G076602 [86]. Additionally, the MYB family regulates the expression profiles of a large number of stress-responsive genes, such as the *OsMYB2* rice gene, whose overexpression activates proline synthetase and transporter genes as well as other stress-related genes [87]. In waterlogging-sensitive and tolerant Chrysanthemum cultivars, the TFs of the ERF, bHLH, and MYB families were up-regulated by waterlogging and down-regulated by reoxygenation, although they were more strongly induced by waterlogging in the tolerant cultivar [88]. Our study indicate that these TFs may have important functions in regulating the response to waterlogging stress. Nonetheless, the active responses of these TFs need to be further investigated.

## 4. Materials and Methods

### 4.1. Plant Material and Waterlogging Treatment 

Two waterlogging-tolerant barley genotypes were used in this study, Yerong [29] and Deder2 [89], due to their potential as sources of tolerance for the barley breeding programs. Although Yerong has been previously described as tolerant to waterlogging, we are considering it as moderately-tolerant to waterlogging based on our conditions after observations made in preliminary tests and refer to it accordingly throughout the manuscript. Seeds for Yerong were obtained from the Tasmanian Institute of Agriculture and School of Land and Food while for Deder2 from the Okayama University, Japan. One seed was sown in soil per each of the cone-tainer cells with a dimension of 4 × 14 cm (top diameter × height) in growth chamber conditions (20 ± 2 °C, 16 h photoperiod) and kept at Brandon Research and Development Centre (BDRC), Brandon, Manitoba, Canada. Cones were filled with a sandy-loam textured soil collected from a field site (BRDC experimental station) where water is prone to accumulate frequently, creating excess moisture problems. The soil was sieved to remove coarse materials. To reduce the effects of variation, for each cone the same amount of soil was measured to ensure that the soil was at the same level, and also the seeds were sown at the same depth.

The experiment consisted of four replications for both the control and treatments for each of the two genotypes tested, and each time-point. Plants were watered on demand from germination. Two-week-old seedlings were submitted to waterlogging treatment, which involved keeping the water level above the soil level for the duration of the treatment. Root samples were collected and carefully washed to prevent mechanical damage, with distilled water, and immediately submerged in liquid nitrogen and then stored at −80 °C until further processing. Four biological replicates for each of the waterlogged and control plants were used. The total RNA was isolated from root tissue samples at the time-points 0 and 72 h of treatment for Yerong from both treated and control plants, and at the points 0, 72, and 120 h of treatment for Deder2 from both treated and control plants.

### 4.2. RNA Extraction, Library Preparation, and Sequencing

Root tissue was pulverized in liquid nitrogen using a mortar and pestle, and total RNA was extracted using an RNeasy Plant Mini Kit (Qiagen, Canada) following the manufacturer’s protocol. Four biological replicates of Yerong at 0 and 72 h, and of Deder2 at 0, 72, and 120 h time-points, were subjected to RNA-Seq analysis. The RNA quality and concentration were verified using a NanoDrop 1000 spectrophotometer (ThermoFisher Scientific, Canada) and an Agilent 2100 Bioanalyzer (Agilent Technologies, Canada) prior to sequencing. The RNA samples were sent to Novogene Corporation, USA, for library preparation, RNA-Seq, and primary sequence analysis. Briefly, following the quality control procedures, the mRNA was enriched using oligo(dT) beads then fragmented randomly in fragmentation buffer, followed by cDNA synthesis using random hexamers and reverse transcriptase. After first-strand synthesis, a custom second-strand synthesis buffer was added to generate the second strand by nick-translation. The final cDNA library was completed after a round of purification, terminal repair, A-tailing, ligation of sequencing adapters, size selection, and PCR enrichment. The library concentration was first quantified using a Qubit 2.0 fluorometer (Life Technologies, Carlsbad, CA, USA), and then diluted to 1 ng/μL before checking insert size on an Agilent 2100 Bioanalyzer (Agilent Technologies, Santa Clara, CA, USA) and quantifying to a greater accuracy by quantitative PCR (q-PCR). The sequencing of the prepared libraries was performed on an Illumina HiSeq™ 4000 platform resulting in at least 20 million paired-end 150 bp reads per sample. 

### 4.3. Differential Expression Analysis

Raw RNA-Seq reads were subjected to quality checking and trimming to remove adaptor sequences, contamination, and low-quality reads. The publicly available Morex genome assembly (International Barley Genome Sequencing Consortium, 2012) was accessed through Ensembl Plants (release 40) and the PE clean reads of each sample were aligned to the assembly using HISAT [90]. Mismatches of no more than two bases were allowed in the alignment. Gene expression level was estimated by transcript abundance, by counting the reads that mapped to genes or exons. After filtering the unknown, low-quality, and adaptor-containing reads, a minimum of 45 million clean reads were obtained for each sample group average (Table S4). Approximately 90% of the clean reads (out of 88.5%–90.4% of total reads) were mapped uniquely to the reference genome (Table S5), and samples displayed high correlation among biological replicates (Figure S2).

To make gene expression data comparable across different genes and experiments, the fragments per kilobase per million fragments mapped (FPKM) method [91] was used with the HTSeq software using the union model. The FPKM values with variance > 100 were used for sample cluster analysis. The readcount values were used in the differential expression analysis and tests for pairwise differential expression were performed by the DESeq R package (1.18.0) with 4 biological replicates to identify the DEGs, with a threshold set as adjusted *p* < 0.05. Genes with an adjusted *p*-value < 0.05 found by DESeq were assigned as differentially expressed. The data discussed in this publication have been deposited in NCBI’s Gene Expression Omnibus [92] and are accessible through GEO Series accession number GSE144077 (https://www.ncbi.nlm.nih.gov/geo/query/acc.cgi?acc=GSE144077). Venn diagrams were created using VennPlex to compare DEGs between the two genotypes, Yerong and Deder2, and between the two different time-points in Deder2 [93].

### 4.4. Quantitative Real-Time PCR (qRT-PCR) Validation 

To validate the repeatability and reproducibility of gene expression data obtained by RNA-Seq in Yerong and Deder2, we performed qRT-PCR on DEGs related to waterlogging-responsive pathways, including ethylene responsive pathways (*ErTF1*), pH regulation during O_2_ deprivation (*GluD1*), anaerobic metabolism (*Hg1*), cell wall thickness (*WAT1*), and cell wall construction (*XEH2*). The qPCR primers for the DEGs were designed and tested for their quality using melt curve analysis and by determining their respective PCR amplification efficiency by a series of 10-fold dilutions of cDNAs. Each assay was optimized so that the efficiency ranged between 95% and 105%, with a coefficient of determination (*R*^2^) > 0.98 (Table S6). After DNase I (Invitrogen) treatment, cDNA was synthesized by assembling in a 20 μL reaction 1X qScript cDNA Supermix (QuantaBio) with 1 μg of total RNA. The reverse transcription PCR was performed in a C1000 Touch™ Thermal Cycler (BioRad Laboratories Inc.) by 25 cycles of amplification as follows: 5 min at 20 °C, 30 min at 42 °C, and 5 min at 85 °C. The qRT-PCR was performed on a QuantStudio 3 (Applied Biosystems) for Yerong samples and StepOne Plus thermal cycler (Applied Biosystems) for Deder2 samples, as follows: Initial denaturation at 95 °C for 3 min, followed by 40 cycles at 95 °C for 15 s, 60 °C for 40 s at which point data was collected, and 72 °C for 45 s, and a final extension at 72 °C for 5 min. Each reaction consisted of 20 μL containing 10 μL of 1X PerfeCTa^®^ SYBR^®^ Green SuperMix (LOW ROX™ mix for Yerong samples and ROX™ mix for Deder2 samples; Quantabio), 4 µL of cDNA diluted by a factor of 10, and 0.3 pM of each primer. Four biological replicates and three technical replicates were included for each experiment. The mRNA expression level was normalized using the reference genes *ELF1-α*, *α-tubulin*, and *β-tubulin* as internal controls (Table S6). The relative expression levels of candidate genes were determined using the 2^−ΔΔCT^ method [94]. To determine if there was a significant difference between the control and treatment groups, a t-test was used assuming a level of significance of 0.05. Pearson’s correlation coefficient was calculated to assess the correlation between the different platforms.

### 4.5. Pathway Enrichment Analysis 

GO enrichment analysis was conducted using g:Profiler [95]. Enriched GO terms were visualized using the Enrichment Map plugin [96] in Cytoscape v.3.7.1 [97] based on the protocol by Reimand et al. [98]. GO terms with more than 500 genes were filtered out to remove the less-specific terms (e.g., catalytic activity).

## 5. Conclusions

**Figure 6:**
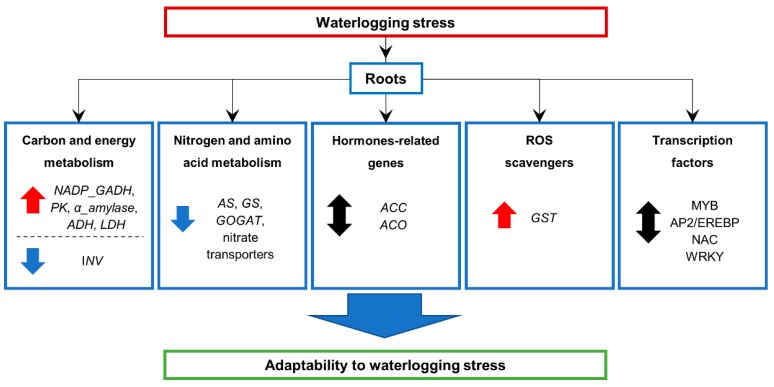
Schematic diagram of the main waterlogging stress responses in the roots of moderately-tolerant (72 h) and tolerant Deder2 (72 and 120 h) barley genotypes. *NADP-GADH*, NADP-dependent glyceraldehyde-3-phosphate dehydrogenase; *PK*, pyruvate kinase; *ADH*, alcohol dehydrogenase; *LDH*, lactate dehydrogenase; *INV*, acid β-fructofuranosidase; *AS*, asparagine synthetase; *GS*, glutamine synthetase; *GOGAT*, glutamate synthase; *ACC*, 1-aminocyclopropane-1-carboxylic acid synthase; *ACO*, 1-aminocyclopropane-1-carboxylic acid (ACC) synthase and *ACC* oxidase; *GST*, glutathione S-transferase; MYB, myeloblastosis; AP2/EREBP, APETALA2/Ethylene-responsive Element Binding Proteins; NAC, this acronym is derived from three genes that were initially discovered to contain a particular domain (the NAC domain): *NAM* (for no apical meristem), *ATAF1* and −2, and *CUC2* (for cup-shaped cotyledon); WRKY, this TF binds a specific promoter sequence of the target gene, known as a W-box and the WRKY proteins contain one or two DNA binding domains of 60 amino acids containing the conserved heptapeptide WRKYGQK. Blue arrow indicates decreased expression, red arrow indicates increased expression, and black arrow indicates down-and up-regulation in Yerong roots at 72 h and Deder2 roots at 72 and 120 h of waterlogging treatment.

This research provides in depth details of gene expression in roots assessed at the early vegetative growth stage (seedling stage) and at specific time-points (72 and 120 h of waterlogging). Our study points out to the roles of several genes involved in different pathways, such as carbon and energy metabolism, nitrogen and amino acid metabolism, hormones-related genes, ROS scavengers, and TFs in response to waterlogging stress in barley (Figure 6). Our results indicated that waterlogging stress down-regulated many genes related to starch and sucrose metabolism, and nitrogen and amino acid metabolism, which is in agreement with other studies in rice [35], maize [36], soybean [39], and cucumber [68]. Conversely, although the sucrose metabolism was down-regulated in rice, starch metabolism was up-regulated [35]. Waterlogging stress also up-regulated many genes related to stress tolerance, including glycolysis and fermentation-related genes, as well as ERFs. These gene expression responses were common across different crops, such as cotton [38,78], poplar [37], rice [35], maize [36,45], soybean [39], and cucumber [68]. We showed that waterlogging tolerance in the barley genotypes tested is potentially characterized by the up-regulation of genes such as *NADP-GADH*, *α-amylase*, *ADH*, *LDH*, and *GST*; while down-regulated genes such as *INV*, *GS*, *AS*, and *GOGAT* are also contributing. The barley waterlogging tolerance model proposed by Luan et al. [44], based on a proteomics approach, highlights the up-regulation in waterlogged roots of several genes, including *PDC*, *ACO*, and *GST*. The comparison with our findings, based on genome-wide analysis of gene expression, shows GST as a common element, indicating that its activity under waterlogging could be an important mechanism in the overall barley resistance to waterlogging stress. Other genes that might be important contributors to waterlogging tolerance in barley are Trichome birefringence, α/β-Hydrolases, Xylanase inhibitor, MATE efflux, serine carboxypeptidase, and SAUR-like auxin-responsive protein, which showed the highest up-regulation in Deder2, increasing with the duration of the waterlogging stress. Overall, our study provides insights into understanding the molecular mechanisms underlying the response to waterlogging in barley. However, further work is needed in order to deeply understand and interpret these responses. These initial findings will be useful in future studies of molecular responses to waterlogging stress and will facilitate genetic research and breeding of barley to improve its waterlogging tolerance.

## Supplementary Materials

The following are available online at https://www.mdpi.com/2223-7747/9/2/240/s1, Figure S1: RNA-Seq and qRT-PCR validation data of five genes, Figure S2: Pearson correlation coefficient matrices, indicated by the 4 × 4 darker blue boxes, showing relationships between samples, Table S1: List of top highly expressed differentially expressed genes (logFC ≥ ±4 and adjusted *p* < 0.05) in the roots of Deder2 after 72 and 120 h of waterlogging, Table S2: Selected differentially expressed genes with altered expression in roots of waterlogged Yerong (72 h) and Deder2 (72 and 120 h) seedlings that are involved in major metabolism pathways, Table S3: Selected differentially expressed genes with altered expression in roots of waterlogged Yerong (72 h) and Deder2 (72 and 120 h) seedlings that are involved in hormones-related genes and reactive oxygen species (ROS) production pathways, Table S4: Summary of sequence assembly reads after Illumina sequencing (average of 4 biological replicates), Table S5: Overview of mapping status by RNA-Seq (average of 4 biological replicates), Table S6: Primer sequences and amplification efficiency for five target waterlogging related genes and three reference genes.
