# Supplementary material for: Genome-Wide Analysis of Gene Expression Provides New Insights into Waterlogging Responses in Barley (Hordeum vulgare L.)
Source: Plants (Basel). 2020 Feb 13;9(2):240. doi: 10.3390/plants9020240 (PMC7076447; doi:10.3390/plants9020240)
Supplement: Supplementary file 1 [file plants-09-00240-s001.pdf]

Supplementary information

Supplementary Figures

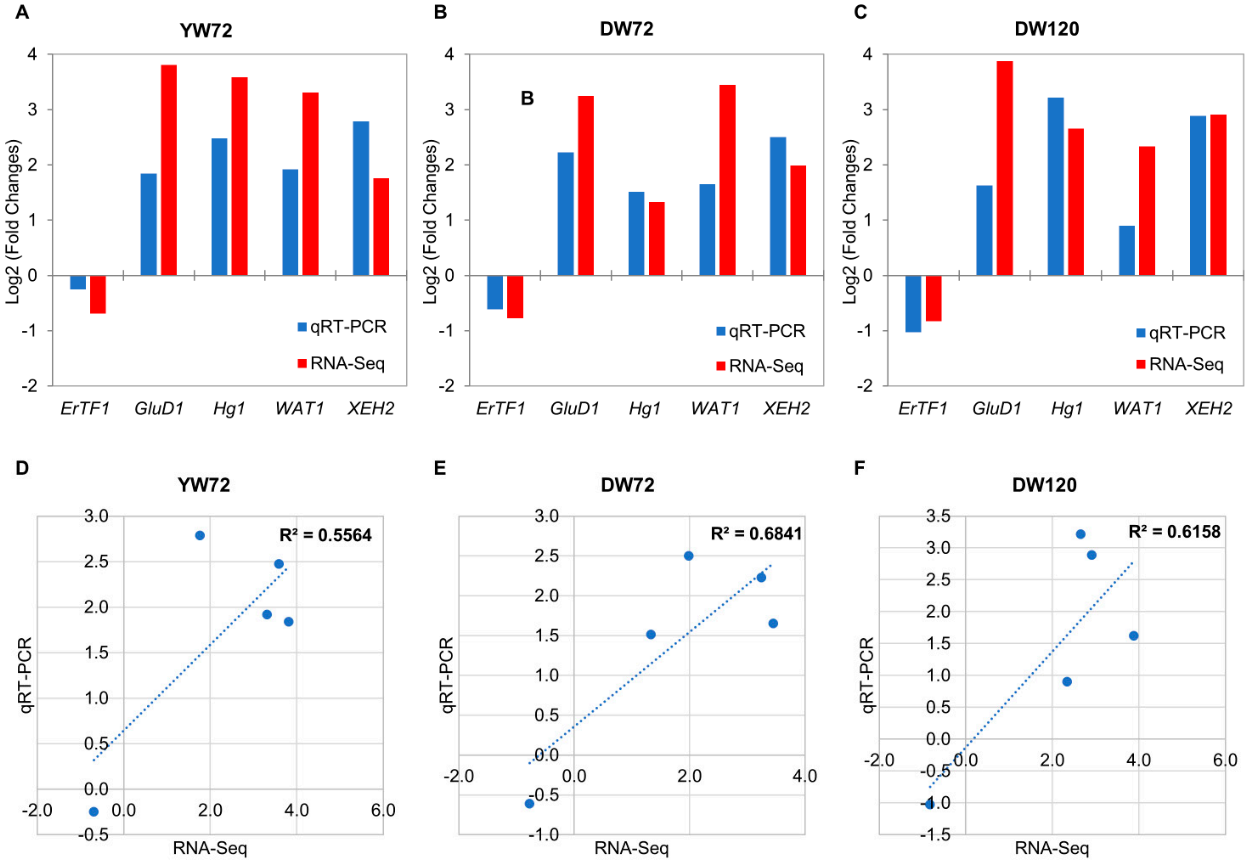

Figure S1. RNA-Seq and qRT-PCR validation data of five genes. Each log_2 ratio of fold changes calculated from qRT-PCR was compared with the log_2 FC of the RNA-Seq data. Four biological replicates were included in each condition. Log_2 fold changes of expression value as measured by RNA-seq and qRT-PCR in Yerong at 72 h of waterlogging (**A**), and Deder2 at 72 h (**B**) and 120 h (**C**), the y-axis indicates the log_2 fold change relative to control. Correlation of the log_2 fold change between RNA-Seq (x-axis) and qRT-PCR (y-axis) in Yerong at 72 h of waterlogging (**D**), and Deder2 at 72 h (**E**) and 120 h (**F**). Each value denotes the mean relative level of expression of four biological replicates. YW72, Yerong 72 h of waterlogging; DW72, Deder2 72 h waterlogging; DW120, Deder2 120 h waterlogging.

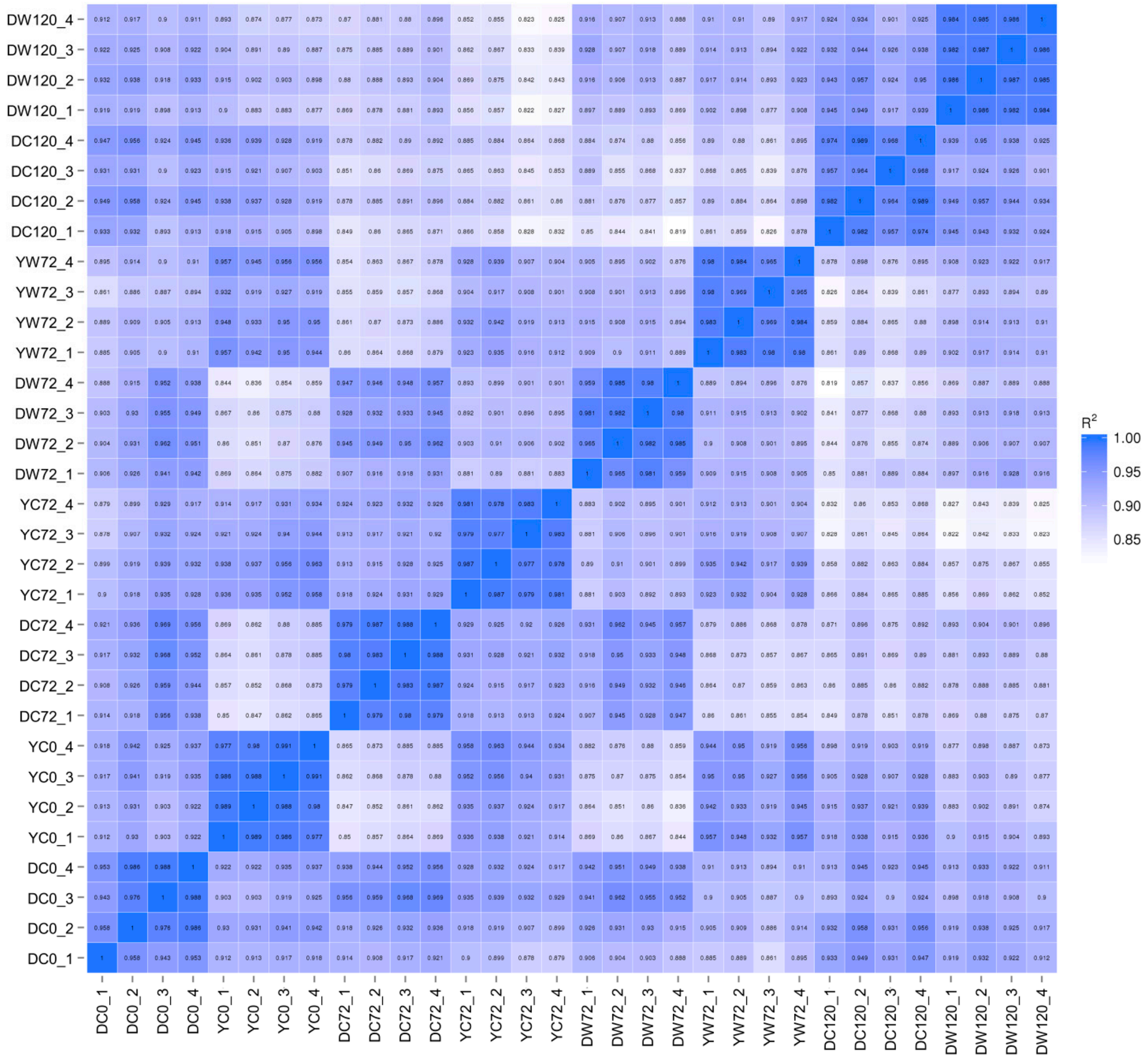

Figure S2. Pearson correlation coefficient matrices, indicated by the 4 x 4 darker blue boxes, showing relationships between samples. Darker blue indicates greater similar expression pattern. R^2 square of the Pearson coefficient. YC0, Yerong 0 h control; YC72, Yerong 72 h control; YW72, Yerong 72 h of waterlogging; DC0, Deder2 0 h control; DC72, Deder2 72 h control; DC120, Deder2 120 h control; DW72, Deder2 72 h waterlogging; DW120, Deder2 120 h waterlogging.

Supplementary tables

Table S1. List of top highly expressed differentially expressed genes (logFC ≥ ±4 and adjusted $p < 0.05$) in the roots of Deder2 after 72 and 120 h of waterlogging.

Gene ID	Gene description	Expression (logFC)	
		72 h	120 h
**Highly up-regulated DEGs**			
HORVU4Hr1G090420	TRICHOME BIREFRINGENCE-LIKE 19	7.47	8.47
HORVU3Hr1G087040	alpha/beta-Hydrolases superfamily protein	4.89	8.23
HORVU6Hr1G036690	unknown function	4.88	8.02
HORVU2Hr1G006130	Xylanase inhibitor protein 1	5.72	7.90
HORVU2Hr1G006170	Xylanase inhibitor protein 2	5.66	7.78
HORVU0Hr1G022350	MATE efflux family protein	4.59	7.38
HORVU6Hr1G012790	serine carboxypeptidase-like 51	6.61	7.00
HORVU7Hr1G107370	SAUR-like auxin-responsive protein family	5.74	6.83
HORVU3Hr1G069210	NifS-like protein	4.23	6.79
HORVU4Hr1G002550	SAUR-like auxin-responsive protein family	Inf	6.41
HORVU1Hr1G088620	Xylanase inhibitor	5.01	6.36
HORVU5Hr1G072650	Calcium-binding EF-hand family protein	5.40	6.27
HORVU4Hr1G085220	unknown function	Inf	5.86
HORVU7Hr1G110900	Ethylene-responsive transcription factor 9	Inf	5.84
HORVU4Hr1G085210	Disease resistance protein RGA2	Inf	5.73
HORVU5Hr1G073710	Protein of unknown function	4.79	5.56
HORVU5Hr1G012290	Glucan 1	4.35	5.55
HORVU2Hr1G090750	Alpha-amylase/subtilisin inhibitor	4.10	5.42
HORVU2Hr1G092510	COBRA-like protein 7	4.78	5.38
HORVU5Hr1G021390	Pathogenesis-related thaumatin superfamily protein	4.76	5.24
HORVU7Hr1G121090	SPX domain gene 3	4.73	5.05
HORVU4Hr1G010940	Eukaryotic translation initiation factor 3 subunit G	4.94	5.04
HORVU2Hr1G016100	poly(A) polymerase 3	4.33	5.01
HORVU4Hr1G000420	Receptor-like protein kinase	4.89	4.56
HORVU1Hr1G086780	undescribed protein	4.59	4.39
HORVU2Hr1G068430	GDSL esterase/lipase	Inf	4.32
HORVU4Hr1G005490	unknown function	4.45	4.27
HORVU2Hr1G106620	Leucine-rich repeat receptor-like protein kinase family protein	5.69	4.05
**Highly down-regulated DEGs**			
HORVU5Hr1G082390	Copalyl diphosphate synthase 2	-4.18	-7.34
HORVU7Hr1G018880	O-methyltransferase family protein	-4.07	-6.84
HORVU5Hr1G092100	Dehydrin	-4.69	-5.68
HORVU4Hr1G081390	transcription activator-related	-4.81	-5.12
HORVU1Hr1G000920	Dirigent protein 21	-4.36	-4.76
HORVU5Hr1G102620	Amaranthin-like lectin	-4.81	-4.58
HORVU4Hr1G001800	Inter-alpha-trypsin inhibitor heavy chain H3	-4.23	-4.13
HORVU2Hr1G102260	cytochrome P450	-5.35	-4.02

*p*adj, adjusted *p*-values; Inf, Infinity due to no transcripts detected in control samples.

Table S2. Selected differentially expressed genes with altered expression in roots of waterlogged Yerong (72 h) and Deder2 (72 and 120 h) seedlings that are involved in major metabolism pathways.

Gene ID	Gene description	YW72 logFC	DW72 logFC	DW120 logFC
**Starch and sucrose metabolism**				
HORVU7Hr1G000260	Acid beta-fructofuranosidase	-1.21	-	-1.60
HORVU7Hr1G000250	Acid beta-fructofuranosidase	-1.21	-	-1.26
HORVU7Hr1G001040	Acid beta-fructofuranosidase	-1.82	-	-
HORVU7Hr1G001070	Acid beta-fructofuranosidase	-2.66	-	-
HORVU2Hr1G091990	Acid beta-fructofuranosidase	-3.41	-1.19	-2.51
HORVU2Hr1G091910	Acid beta-fructofuranosidase	-3.76	-1.24	-1.60
HORVU2Hr1G090750	Alpha-amylase/subtilisin inhibitor	7.94	4.10	5.42
HORVU7Hr1G091250	alpha-amylase-like	1.70	1.57	1.10
HORVU3Hr1G067620	alpha-amylase-like 3	-1.27	-1.00	-
HORVU5Hr1G019030	ATP-dependent 6-phosphofructokinase	2.50	-	2.83
HORVU3Hr1G019580	ATP-dependent 6-phosphofructokinase 3	-	-	1.62
HORVU1Hr1G075550	ATP-dependent 6-phosphofructokinase 3	1.14	-	2.22
HORVU7Hr1G047000	ATP-dependent 6-phosphofructokinase 3	-1.09	-	-
HORVU1Hr1G056180	D-3-phosphoglycerate dehydrogenase	-1.26	-1.51	-2.76
HORVU4Hr1G019570	fructose-bisphosphate aldolase 2	1.55	-	1.79
HORVU3Hr1G088500	fructose-bisphosphate aldolase 2	1.53	1.23	1.07
HORVU3Hr1G088540	fructose-bisphosphate aldolase 2	1.05	0.66	0.94
HORVU5Hr1G032980	fructose-bisphosphate aldolase 2	-1.24	-	-1.40
HORVU7Hr1G115590	sucrose phosphate synthase 1F	1.33	-	0.64
HORVU7Hr1G007220	sucrose synthase 1	-1.27	-0.95	-
HORVU7Hr1G033230	sucrose synthase 4	1.66	1.08	1.93
HORVU7Hr1G120520	sucrose synthase 6	-1.07	-	-
**Glycolysis**				
HORVU1Hr1G018540	alanine aminotransferase 2	2.81	1.09	2.70
HORVU4Hr1G082700	glyceraldehyde-3-phosphate dehydrogenase C2	2.32	-	-
HORVU7Hr1G074690	glyceraldehyde-3-phosphate dehydrogenase C2	1.59	0.62	1.14
HORVU6Hr1G054520	glyceraldehyde-3-phosphate dehydrogenase C2	1.54	1.18	1.25
HORVU2Hr1G036110	NADP-dependent glyceraldehyde-3-phosphate dehydrogenase	-	2.72	3.14
HORVU0Hr1G005720	Pyruvate kinase family protein	2.22	-	1.24
HORVU2Hr1G040570	Pyruvate kinase family protein	2.12	-	1.29
HORVU2Hr1G119460	Pyruvate kinase family protein	1.86	-	1.29
HORVU5Hr1G041120	Pyruvate kinase family protein	1.79	1.29	1.92
HORVU1Hr1G054380	Pyruvate kinase family protein	-1.34	-1.05	-0.66
**Fermentation**				
HORVU0Hr1G012860	Alcohol dehydrogenase	-	1.32	-2.39
HORVU5Hr1G062030	Alcohol dehydrogenase	-1.30	-	-
HORVU0Hr1G012940	Alcohol dehydrogenase	-2.03	-	-0.91
HORVU7Hr1G090560	Alcohol dehydrogenase	-3.10	-	-
HORVU6Hr1G087220	Alcohol dehydrogenase	-3.64	-1.37	-2.95
HORVU1Hr1G082250	alcohol dehydrogenase 1	4.53	3.14	5.26
HORVU3Hr1G034820	alcohol dehydrogenase 1	3.56	-	3.23
HORVU1Hr1G003130	alcohol dehydrogenase 1	2.97	-	-
HORVU4Hr1G016770	alcohol dehydrogenase 1	2.91	0.91	2.56
HORVU2Hr1G020900	alcohol dehydrogenase 1	2.70	1.57	2.94
HORVU5Hr1G010490	alcohol dehydrogenase 1	2.61	-	2.29

Gene ID	Gene description	YW72 logFC	DW72 logFC	DW120 logFC
HORVU4Hr1G016780	alcohol dehydrogenase 1	2.45	1.47	4.27
HORVU0Hr1G010220	alcohol dehydrogenase 1	2.34	-	1.66
HORVU0Hr1G008870	alcohol dehydrogenase 1	2.26	-	2.21
HORVU4Hr1G016810	alcohol dehydrogenase 1	2.19	-	2.47
HORVU2Hr1G010010	alcohol dehydrogenase 1	-1.46	-1.10	-2.07
HORVU2Hr1G060010	L-lactate dehydrogenase	-	2.31	-
HORVU7Hr1G096800	L-lactate dehydrogenase A	-	-	3.11
HORVU6Hr1G015500	L-lactate dehydrogenase A	2.68	-	1.57
HORVU2Hr1G012830	L-lactate dehydrogenase B	-	2.99	4.74
HORVU2Hr1G012830	L-lactate dehydrogenase B	4.34	2.99	4.74
HORVU2Hr1G012660	pyruvate decarboxylase-2	-	-	-4.11
HORVU4Hr1G056050	pyruvate decarboxylase-2	1.35	-	1.30
HORVU3Hr1G018650	pyruvate decarboxylase-2	-1.16	-1.05	-2.54
**Nitrogen and amino acid metabolism**				
HORVU4Hr1G056240	Asparagine synthetase [glutamine-hydrolyzing] 1	-2.53	-3.04	-2.36
HORVU2Hr1G044560	Ferredoxin-dependent glutamate synthase 2	-1.56	-1.76	-
HORVU1Hr1G038060	Glutamine synthetase	-0.64	-	-
HORVU6Hr1G074030	glutamine synthetase 1;4	-0.80	-1.00	-0.66
HORVU6Hr1G005580	high affinity nitrate transporter 2.6	-	-	-1.54
HORVU6Hr1G005590	high affinity nitrate transporter 2.6	-1.34	-	-2.06
HORVU6Hr1G005930	high affinity nitrate transporter 2.6	-1.97	-	-
HORVU6Hr1G005600	high affinity nitrate transporter 2.6	-2.10	-1.27	-3.16
HORVU6Hr1G005770	high affinity nitrate transporter 2.6	-2.37	-1.00	-1.35
HORVU6Hr1G005780	high affinity nitrate transporter 2.6	-5.22	-1.63	-4.04
HORVU6Hr1G053710	High-affinity nitrate transporter 3.1	-0.71	-	-0.69
HORVU5Hr1G115500	High-affinity nitrate transporter 3.1	-1.88	-	-

YW72, Yerong 72 h of waterlogging; DW72, Deder2 72 h waterlogging; DW120, Deder2 120 h waterlogging; Inf, Infinity due to no transcripts detected in control samples.

Table S3. Selected differentially expressed genes with altered expression in roots of waterlogged Yerong (72 h) and Deder2 (72 and 120 h) seedlings that are involved in hormones-related genes and reactive oxygen species (ROS) production pathways.

Gene ID	Gene description	YW72 logFC	DW72 logFC	DW120 logFC
**Hormones-related genes**				
HORVU1Hr1G020410	1-aminocyclopropane-1-carboxylate oxidase	-	1.31	-
HORVU6Hr1G079640	1-aminocyclopropane-1-carboxylate oxidase	-4.49	-2.10	-2.50
HORVU4Hr1G017660	1-aminocyclopropane-1-carboxylate oxidase	-1.65	-1.71	-2.77
HORVU6Hr1G079600	1-aminocyclopropane-1-carboxylate oxidase	-6.66	-2.08	-3.35
HORVU7Hr1G086650	1-aminocyclopropane-1-carboxylate oxidase 1	-	-1.56	-
HORVU5Hr1G067490	1-aminocyclopropane-1-carboxylate oxidase 1	3.88	3.04	2.36
HORVU7Hr1G116830	1-aminocyclopropane-1-carboxylate oxidase 1	-	-	2.29
HORVU5Hr1G067530	1-aminocyclopropane-1-carboxylate oxidase 1	2.51	2.34	2.02
HORVU2Hr1G094230	1-aminocyclopropane-1-carboxylate synthase 11	1.58	2.31	-
HORVU4Hr1G009800	1-aminocyclopropane-1-carboxylate synthase 11	-3.78	-1.10	-
HORVU2Hr1G122970	Auxin-induced protein 5NG4	-	-3.16	-8.71
HORVU2Hr1G022640	Auxin-induced protein-like	-1.59	-1.44	-2.87
HORVU5Hr1G093580	Auxin-responsive protein IAA30	1.15	1.18	1.29
HORVU7Hr1G107370	SAUR-like auxin-responsive protein family	4.68	5.74	6.83
HORVU5Hr1G085680	SAUR-like auxin-responsive protein family	4.65	1.97	4.78
HORVU4Hr1G002550	SAUR-like auxin-responsive protein family	4.04	Inf	6.41
HORVU3Hr1G095580	SAUR-like auxin-responsive protein family	4.01	0.89	1.43
HORVU7Hr1G107360	SAUR-like auxin-responsive protein family	3.31	3.62	1.56
HORVU7Hr1G017790	SAUR-like auxin-responsive protein family	2.24	-	2.12
HORVU7Hr1G107270	SAUR-like auxin-responsive protein family	1.65	2.81	-
HORVU5Hr1G086240	SAUR-like auxin-responsive protein family	1.56	1.29	-
HORVU6Hr1G080050	SAUR-like auxin-responsive protein family	1.47	2.24	-
HORVU6Hr1G027640	SAUR25-auxin-responsive SAUR family member	1.17	0.97	-
HORVU5Hr1G062580	SAUR-like auxin-responsive protein family	1.09	0.72	-
HORVU7Hr1G091350	SAUR-like auxin-responsive protein family	-0.86	-1.45	-1.06
HORVU5Hr1G076690	SAUR-like auxin-responsive protein family	-1.16	-0.90	-
HORVU6Hr1G080000	SAUR-like auxin-responsive protein family	-1.27	0.81	-
HORVU7Hr1G096870	SAUR-like auxin-responsive protein family	-1.39	-	-
HORVU2Hr1G092070	SAUR-like auxin-responsive protein family	-2.19	-0.65	-
HORVU4Hr1G002600	SAUR-like auxin-responsive protein family	-2.58	-	-
HORVU7Hr1G084790	SAUR-like auxin-responsive protein family	-	2.62	-
HORVU4Hr1G002570	SAUR-like auxin-responsive protein family	-	-	2.58
HORVU2Hr1G110310	SAUR-like auxin-responsive protein family	-	-	1.69
HORVU6Hr1G020840	SAUR-like auxin-responsive protein family	-	1.61	-
HORVU7Hr1G096880	SAUR-like auxin-responsive protein family	-	-	1.16
HORVU2Hr1G110430	SAUR-like auxin-responsive protein family	-	-2.16	-
HORVU1Hr1G076730	gibberellin 2-oxidase	-	-1.88	-1.73
HORVU3Hr1G022840	gibberellin 3-oxidase 1	-	-2.78	-4.48
HORVU2Hr1G034860	Gibberellin-regulated family protein	-	1.65	1.09
HORVU4Hr1G052880	AP2-like ethylene-responsive transcription factor	1.83	1.53	-
HORVU5Hr1G045290	AP2-like ethylene-responsive transcription factor	-0.90	-1.65	-0.98
HORVU2Hr1G018830	Ethylene-responsive transcription factor 1	3.51	1.45	3.76
HORVU2Hr1G094810	Ethylene-responsive transcription factor 1	3.05	1.75	-
HORVU5Hr1G080790	Ethylene-responsive transcription factor 1	2.47	-	-1.79

Gene ID	Gene description	YW72 logFC	DW72 logFC	DW120 logFC
HORVU1Hr1G064030	Ethylene-responsive transcription factor 1	2.18	2.71	-
HORVU1Hr1G058940	Ethylene-responsive transcription factor 1	1.86	-	1.34
HORVU0Hr1G018280	Ethylene-responsive transcription factor 1	1.31	1.25	-
HORVU1Hr1G090250	Ethylene-responsive transcription factor 1	-	2.35	-
HORVU4Hr1G023110	Ethylene-responsive transcription factor 1	-	1.84	-
HORVU5Hr1G068450	Ethylene-responsive transcription factor 1	-	1.82	-1.69
HORVU1Hr1G063100	Ethylene-responsive transcription factor 1	-	1.06	-
HORVU6Hr1G074970	Ethylene-responsive transcription factor 1	-	-1.01	-
HORVU4Hr1G071890	Ethylene-responsive transcription factor 1	-	-1.71	-1.42
HORVU4Hr1G077360	Ethylene-responsive transcription factor 1	-2.53	-2.38	-1.50
HORVU5Hr1G036590	Ethylene-responsive transcription factor 1	-1.65	-	-2.74
HORVU2Hr1G071270	Ethylene-responsive transcription factor 1	-	-	0.91
HORVU7Hr1G090440	Ethylene-responsive transcription factor 1	-	-	-2.01
HORVU4Hr1G079630	Ethylene-responsive transcription factor 1B	-	Inf	3.47
HORVU2Hr1G103660	Ethylene-responsive transcription factor 1B	-	1.45	
HORVU3Hr1G030310	Ethylene-responsive transcription factor 3	-	1.09	
HORVU0Hr1G016540	Ethylene-responsive transcription factor 4	2.50	1.80	-1.40
HORVU1Hr1G082310	Ethylene-responsive transcription factor 4	1.82	2.30	-
HORVU7Hr1G035440	Ethylene-responsive transcription factor 4	1.78	1.63	-
HORVU7Hr1G026940	Ethylene-responsive transcription factor 5	-2.52	-1.17	-
HORVU2Hr1G037540	Ethylene-responsive transcription factor 8	-2.04	-	1.47
HORVU7Hr1G110900	Ethylene-responsive transcription factor 9	4.91	Inf	5.84
HORVU6Hr1G078520	Ethylene-responsive transcription factor 9	-0.99	1.66	-
HORVU4Hr1G013410	Ethylene-responsive transcription factor 9	-	1.16	-
HORVU6Hr1G025010	Ethylene-responsive transcription factor 9	-		-1.31
HORVU5Hr1G112440	Ethylene-responsive transcription factor 10	-1.43	-1.10	-
HORVU5Hr1G055190	Ethylene-responsive transcription factor 10	-	1.50	-
HORVU7Hr1G088790	Ethylene-responsive transcription factor 11	1.36	1.57	-
HORVU3Hr1G003400	Ethylene-responsive transcription factor 11	-	1.48	-
HORVU3Hr1G026950	Ethylene-responsive transcription factor 11	-	-4.05	-
HORVU3Hr1G090740	Ethylene-responsive transcription factor 13	2.16	2.59	-
HORVU6Hr1G065430	Ethylene-responsive transcription factor 13	-	1.65	-
HORVU6Hr1G078510	Ethylene-responsive transcription factor ERF112	-	1.39	-
HORVU5Hr1G080430	ethylene-responsive element binding factor 13	2.76	2.29	-
**ROS scavengers**				
HORVU5Hr1G104120	ascorbate peroxidase 2	2.61	-	-
HORVU1Hr1G001560	Glutathione S-transferase family protein	-	2.47	4.76
HORVU5Hr1G108660	Glutathione S-transferase family protein	-	1.89	3.78
HORVU3Hr1G107350	Glutathione S-transferase family protein	2.30	2.31	2.71
HORVU1Hr1G021180	Glutathione S-transferase family protein	2.06	1.72	1.99
HORVU2Hr1G095460	Glutathione S-transferase family protein	1.15	1.07	1.98
HORVU4Hr1G082810	Glutathione S-transferase family protein	-	2.81	1.93
HORVU1Hr1G002160	Glutathione S-transferase family protein	-	1.13	1.80
HORVU0Hr1G019300	Glutathione S-transferase family protein	-	1.32	1.71
HORVU7Hr1G002370	Glutathione S-transferase family protein	-	1.35	1.23
HORVU1Hr1G021170	Glutathione S-transferase family protein	-	1.40	1.13
HORVU7Hr1G108570	Glutathione S-transferase family protein	-2.91	-1.60	-1.43
HORVU2Hr1G125200	Peroxidase superfamily protein	2.72	2.56	2.15
HORVU3Hr1G112040	Peroxidase superfamily protein	1.64	2.00	1.48

Gene ID	Gene description	YW72 logFC	DW72 logFC	DW120 logFC
HORVU4Hr1G065000	Peroxidase superfamily protein	1.74	2.21	1.47
HORVU5Hr1G097130	Peroxidase superfamily protein	-	-1.25	-1.06
HORVU1Hr1G066600	Peroxidase superfamily protein	-	-1.01	-1.12
HORVU2Hr1G124970	Peroxidase superfamily protein	-1.66	-1.17	-1.32
HORVU1Hr1G066580	Peroxidase superfamily protein	-	-1.21	-1.63
HORVU1Hr1G066540	Peroxidase superfamily protein	-	-1.50	-1.69
HORVU1Hr1G066610	Peroxidase superfamily protein	-1.05	-1.12	-1.75
HORVU1Hr1G016980	Peroxidase superfamily protein	-1.58	-1.74	-1.77
HORVU7Hr1G020940	Peroxidase superfamily protein	-2.35	-1.21	-1.98
HORVU7Hr1G108530	Peroxidase superfamily protein	-2.19	-1.54	-2.65
HORVU7Hr1G020960	Peroxidase 2	-2.47	-1.31	-2.33
HORVU7Hr1G008390	Fe superoxide dismutase 2	1.19	-	-
HORVU7Hr1G121700	catalase 2	-	-0.61	0.83
HORVU7Hr1G038770	dehydroascorbate reductase 1	1.00	-	1.03
HORVU7Hr1G030810	glutathione peroxidase 1	-1.52	-1.52	1.21
HORVU6Hr1G063830	glutathione peroxidase 6	-1.93	-0.96	-
HORVU4Hr1G073930	glutathione reductase	0.80	-	-
HORVU6Hr1G089780	glutathione reductase	-	-	1.06
HORVU0Hr1G017540	glutathione reductase	-	-	0.94
HORVU5Hr1G076430	L-ascorbate oxidase	1.03	2.50	-
HORVU5Hr1G076510	L-ascorbate oxidase	1.30	2.36	-
HORVU5Hr1G076500	L-ascorbate oxidase	1.14	2.28	-
HORVU5Hr1G057800	L-ascorbate oxidase	-	1.39	-1.47
HORVU7Hr1G087240	L-ascorbate oxidase	-1.87	-	-1.04
HORVU3Hr1G038800	L-ascorbate oxidase-like protein	-1.53	-	-
HORVU2Hr1G096250	L-ascorbate oxidase homolog	-2.58	-	-1.64

YW72, Yerong 72 h of waterlogging; DW72, Deder2 72 h waterlogging; DW120, Deder2 120 h waterlogging; Inf, Infinity due to no transcripts detected in control samples.

Table S4. Summary of sequence assembly reads after Illumina sequencing (average of 4 biological replicates).

Sample	Raw reads	Clean reads	Clean bases	Error rate (%)	Q20 (%)	Q30 (%)	GC content (%)
YC0	54354875	52908323	7.95G	0.02	98.71	96.08	57.33
YC72	52836049	51692383	7.75G	0.02	98.52	95.57	57.59
YW72	53545857	52252451	7.83G	0.02	98.67	95.98	57.32
DC0	57040589	55621298	8.35G	0.02	98.68	95.98	57.31
DC72	51153058	49857241	7.48G	0.02	98.67	96.01	57.43
DC120	49097605	47898069	7.18G	0.02	98.51	95.61	56.93
DW72	47126449	45755388	6.88G	0.02	98.64	95.94	58.17
DW120	56959292	55669368	8.35G	0.02	98.67	95.95	56.41

Raw reads, Statistical data for the original sequences; Clean reads, Calculation method is the same as for raw reads, but the statistics file is the filtered data; Clean bases, sequence number*sequence length (transformed into G bases); Q20, Q30, Percentage of the number of bases of Phred score greater than 20, 30 respectively, GC content, Percentage of G and C bases among the total base number.

Table S5. Overview of mapping status by RNA-Seq (average of 4 biological replicates).

Sample	Total reads	Total mapped	Multiple mapped	Uniquely mapped	Reads map to '+'	Reads map to '-'	Non-splice reads	Splice reads
YC0	52908323	47839328	5130372	42708956	21368926	21340030	28260121	14448836
YC72	51692383	46344140	4740056	41604085	20826693	20777392	27858966	13745119
YW72	52252451	46888644	5119619	41769025	20901112	20867913	27538542	14230482
DC0	55621298	49874617	5374601	44500016	22270800	22229216	30002653	14497363
DC72	49857241	44496380	4692869	39803511	19920525	19882987	26565286	13238226
DC120	47898069	42587330	4717646	37869684	18948559	18921125	24818521	13051163
DW72	45755388	40475882	4350888	36124994	18073201	18051793	24935080	11189914
DW120	55669368	49795509	5592038	44203471	22112912	22090559	28242552	15960919

Total reads, Count number of filtered reads (Clean data); Total mapped Total number of reads could be mapped to the genome; Multiple mapped, Count number of reads that could be mapped to multiple sites in the reference genome; Uniquely mapped, Count number of reads that can be uniquely mapped to the reference genome; Reads map to '+', Reads map to '-', Count number of reads that mapped to positive strand or minus strand, respectively; Splice reads, Count number of reads that could be segmented and mapped to 2 exons, also named junction reads.

Table S6. Primer sequences and amplification efficiency for five target waterlogging related genes and three reference genes.

Gene Name (Primer ID)	Tentative annotation	Primer Sequences (5' to 3')	AE (%)
Ethylene-responsive transcription factor 1 (*ErTF1*)	Ethylene biosynthesis	F: TCGACGAGGACTCTTCTGTG<br>R: GGGTAGCTGGAACATCATGAAC	94.72
Glutamate decarboxylase (*GluD1*)	pH regulation during O_2 deprivation	F: CATGGCCTCCATCAACAAGAAC<br>R: ACGCACCGGTTCTGGAG	98.52
Non-symbiotic hemoglobin (*Hg1*)	Anaerobic metabolism	F: TGCGCAGTTGCGGAAAG<br>R: AACCGCGTCACCTCGAA	97.38
WAT1-related protein (*WAT1*)	Cell wall thickness	F: TAGGAATGGAGTCCTTGAAACT<br>R: CACTGGCACAAAGCACTATAC	97.92
Xyloglucan endotransglucosylase hydrolase (*XEH2*)	Cell wall construction	F: TCTACCAGGACGTGGACAT<br>R: CAGGTACTGGTTCTTGGACTG	98.70
Elongation factor 1-α (*ELF1-α*) – reference gene		F: CACACCTCACACATTGCTGTC<br>R: ACCAGCATCACCATTCTTGAG	101.37
α-Tubulin (*α-tubulin*) – reference gene		F: CTTGACCGTATCAGGAAGCTG<br>R: AGAAGAGAACCAAGGCCAGAG	99.53
β-Tubulin (*β-tubulin*) – reference gene		F: GGAGGCTGAGAACTGTGACTG<br>R: TCAGGGTACTCCTCCCTGATT	92.80

F, Forward; R, Reverse; AE, Amplification efficiency.
